# Identification and characterization of circRNAs in *Pyrus betulifolia* Bunge under drought stress

**DOI:** 10.1371/journal.pone.0200692

**Published:** 2018-07-17

**Authors:** Jinxing Wang, Jing Lin, Hong Wang, Xiaogang Li, Qingsong Yang, Hui Li, Youhong Chang

**Affiliations:** Institute of Pomology, Jiangsu Academy of Agricultural Sciences/Jiangsu Key Laboratory for Horticultural Crop Genetic Improvement, Nanjing, China; Nanjing Agricultural University, CHINA

## Abstract

Circular RNAs (circRNAs) play important roles in miRNA function and transcriptional control. However, little is known regarding circRNAs in the pear. In this study, we identified circRNAs using deep sequencing and analyzed their expression under drought stress. We identified 899 circRNAs in total, among which 33 (23 upregulated, 10 downregulated) were shown to be dehydration-responsive. We performed GO and KEGG enrichment analysis to predict the functions of differentially expressed circRNAs. 309 circRNAs were predicted to act as sponges for 180 miRNAs. A circRNA-miRNA co-expression network was constructed based on correlation analysis between the differentially expressed circRNAs and their miRNA binding sites. Our study will provide a rich genetic resource for the discovery of genes related to drought stress, and can readily be applied to other fruit trees.

## Introduction

According to the recent research, eukaryotic genomes encode a large number of ncRNAs (non-coding RNAs) [1,2]. ncRNAs have no or little protein-coding potential, but play roles in various biological processes [3]. Circular RNA (circRNA) is an ncRNA molecule that is devoid of the 5ʼ cap and poly A tail. circRNA is a ring structure formed by a covalent bond, and circRNAs were first discovered in yeast [4] and humans [5] in 1980 and 1993, respectively. Circular RNAs are generated during splicing through various mechanisms [6]: most circular RNAs are created from back-spliced exons, while others originate from introns [7]. circRNAs are more stable than linear RNA molecules in cells, have a longer half-life, and can resist RNAase R degradation [8]. According to their general location in the genome, there are five types of circRNAs: exonic circRNAs, intronic circRNAs, sense overlapping circRNAs, antisense circRNAs, and intergenic circRNAs [9].

CircRNAs are ubiquitous among all domains of life, and can fulfill a diverse range of biological functions. They can serve as competing endogenous RNAs to bind micro RNAs (miRNAs) [10,11], gene transcription and expression regulators [12], and RNA binding proteins (protein sponges) [13]. For example, more than 70 miRNA target sites (selectively conserved) are present at ciRS-7 and ciRS-7 is stongly linked with Argonaute protein in an miR-7-dependent style in humans [10]. circRNAs also play vital roles in the stress response. For instance, 163 circRNAs were significantly differentially expressed in tomatoes exposed to cold stress compared to a control [14]. Aided by the development of high-throughput sequencing platforms and bioinformatics methods, an abundance of circRNAs have recently been identified in Archaea [15], humans, mouse [16], Arabidopsis [17,18], and rice [12].

Pear (*Pyrus* spp.) is among the most important fruits in the world, and its production is strongly affected by drought. As one of the main varieties of pear, the birch-leaf pear (*Pyrus betulifolia* Bunge) exhibits high disease resistance and has a high tolerance of abiotic stresses such as drought and salinity [19]. These qualities make it an important source of valuable drought tolerance genes for improving both fruit quality and tree resistance to drought. Therefore, identifying the drought resistance genes of *P*. *betulifolia* holds great value for molecular breeding efforts.

Several studies have shown that multiple circRNAs are induced by stress. In this study, we used RNA sequencing (RNA-seq) to analyze differentially expressed circRNAs in birch-leaf pear leaves under drought stress. Our results will facilitate the development of pear breeding programs and provide a foundation for the identification of circRNAs in other fruit trees.

## Materials and methods

### Birch-leaf pear samples

Birch-leaf pear (*P*. *betulifolia* Bunge) seedlings were grown in seedling beds at the national germplasm orchard of the Institute of Horticulture of Jiangsu Academy of Agricultural Sciences, Nanjing, Jiangsu, China. Seedlings were placed in a growth chamber under a 24-h cycle: 14 h at 25°C in light and 10 h at 20°C in the dark, as per our previous report [20]. Six-leaf-stage seedlings were inserted into a beaker containing distilled water for 2 d before exposure to dehydration treatment. Seedlings were then transferred into a 1/2 Murashige and Skoog Basal (MS) solution containing 15% polyethylene glycol (PEG) to simulate drought stress. The leaves of control and treatment seedlings were collected at 48 h after treatment in triplicate, rinsed with distilled water, frozen in liquid nitrogen, and stored at –80°C until further use.

### RNA preparation

Total RNA from each of the six samples was extracted using a Takara Mini BEST Plant RNA Extraction Kit following the manufacturer’s protocol. RNA quality was verified using formaldehyde agarose gel electrophoresis, and RNA quantity was determined using a NanoDrop ND-1000 spectrophotometer. A total of 5 μg of RNA per sample was used for the experiment. To obtain ribosomal RNA (rRNA)-depleted RNA, rRNAs were depleted using the Epicentre Ribo-zero rRNA Removal Kit (Epicentre, USA). Subsequently, sequencing libraries were generated from the rRNA-depleted and RNase R-digested RNAs using the NEBNext Ultra Directional RNA Library Prep Kit for Illumina (NEB, USA) following the manufacturer’s recommendations. Finally, the library was purified using the AMPure XP system and qualified using the Agilent Bioanalyzer 2100 system.

### Clustering and sequencing

The clustering of index-coded samples was performed on a cBot Cluster Generation System using the HiSeq PE Cluster Kit v4 cBot (Illumina) according to the manufacturer’s instructions. After cluster generation, the libraries were sequenced on an Illumina Hiseq 4000 platform and 150-bp paired-end reads were generated.

### Quality control

Raw data (raw reads in fastq format) was first processed using a custom Perl script. Clean data (clean reads) were obtained after removing adapter-containing reads, poly-N-containing reads, and low quality reads from the raw data. The Q20, Q30, and GC content of the clean data were calculated. All downstream analysis was based on the clean, high-quality data generated in this step.

### Mapping to reference genome

The reference genome and gene model annotation files were downloaded from the pear genome website [21] (http://peargenome.njau.edu.cn/). The reference genome index was built using Bowtie v2.0.6 software, and paired-end clean reads were aligned to the reference genome using TopHat v2.0.9 software.

### CircRNA identification

We used find_circ version 1.1 [11] and CIRI version 2.0.5 [22] to identify circRNAs. For find_circ, unmapped reads were retained, and 20-mers from 5ʼ and 3ʼ end of these reads were extracted and aligned independently to reference sequences using Bowtie v2.0.6. Anchor sequences were extended by the find_circ algorithm such that the complete read aligned and the breakpoints were flanked by GU/AG splice sites. Next, back-spliced reads with at least two supporting reads were identified as circRNAs. The CIRI algorithm was another tool to identify circRNAs, it scans SAM files twice and collects sufficient information to identify and characterize circRNAs. CIRI was performed with default options, Counts of identified circRNA reads were normalized by read length, and the number of reads mapping was determined after CIRI prediction. Finally, the sequences of intersection by the two approach were identified to be circRNA.

### Quantification

circRNA expression levels were normalized based on transcripts per million (TPM) using the following formula [23]:

normalizedexpression=(mappedreads)/(totalreads)×1,000,000.

### Differential expression

Differential expression between the two groups 0h and 48h) was performed using DESeq2 version 1.6.3 software [24]. P-values were adjusted using the Benjamini and Hochberg method. By default, the corrected p-value threshold for differential expression was set to 0.05.

### GO and KEGG enrichment analysis

Gene Ontology (GO, http://www.geneontology.org/) enrichment analysis for the source genes of differential circRNAs was performed using GOseq software (version 1.18.0). KEGG [25] is a database for understanding the high-level functions of biological systems (http://www.genome.jp/kegg/). The KOBAS web server [26] was used for the Kyoto Encyclopedia of Genes and Genomes (KEGG) pathway enrichment analysis.

### MiRNA binding site prediction

CircRNA could inhibit the function of miRNA by binding to miRNA. In order to further research the function of circRNA, we analysis the miRNA binding site of the identified circRNA using PsRobot software. This software is a web-based easy-to-use tool that used miRNA and circRNA sequence (including junction site) for target gene prediction, and it is performs fast analysis to identify miRNA with stem-loop shaped precursors among batch input data using a modified Smith-Waterman algorithm [27].

### RT-qPCR validation

The expression profiles of drought-responsive circRNAs were validated through quantitative PCR. Total RNA for use as a template was extracted from leaves using the Total RNA Kit (Tiangen, Beijing, China) according to the manufacturer’s instructions, the first cDNA strand was synthesized from 1000ng total RNA in a volume of 20μl using the PrimeScriptTM RT reagent Kit with gDNA eraser (Perfect Real Time), (Clontech, Shiga, Japan), according to the manufacturer’s protocol. Eight circRNAs were randomly selected from the differentially expressed circRNA and analyzed using RT-qPCR. Primers were designed using Primer5 software [28], and RT-qPCR was performed on a 7500 Real-Time PCR System (Applied Biosystems, CA, USA). The total reaction volume was 20 μl, containing 10 μl 2X SYBR Premix Ex Taq™ (TaKaRa Bio Inc., Japan), 1 μl complementary DNA (cDNA) reaction mixture, 0.5 μl of each primer, 0.5 μl ROX Reference DyeII, and 7.5 μl ddH_2_O. Glyceraldehyde-3-phosphate dehydrogenase (GAPDH) gene was used as housekeeping gene for normalization [29]. The primers sequence used in our PCR experiments are described in S1 File. PCR was performed as follows: pre-denaturation at 95°C for 30 s, denaturation at 95°C for 3 s, annealing at 60°C for 30 s, and 55–95°C for melting curve analysis. All reactions were performed using biological triplicates. The 2^-ΔΔCT^ method was used to calculate relative changes in gene expression between control and treatment plants [30].

## Results

### Identification of circRNAs in pear

**Fig 1:**
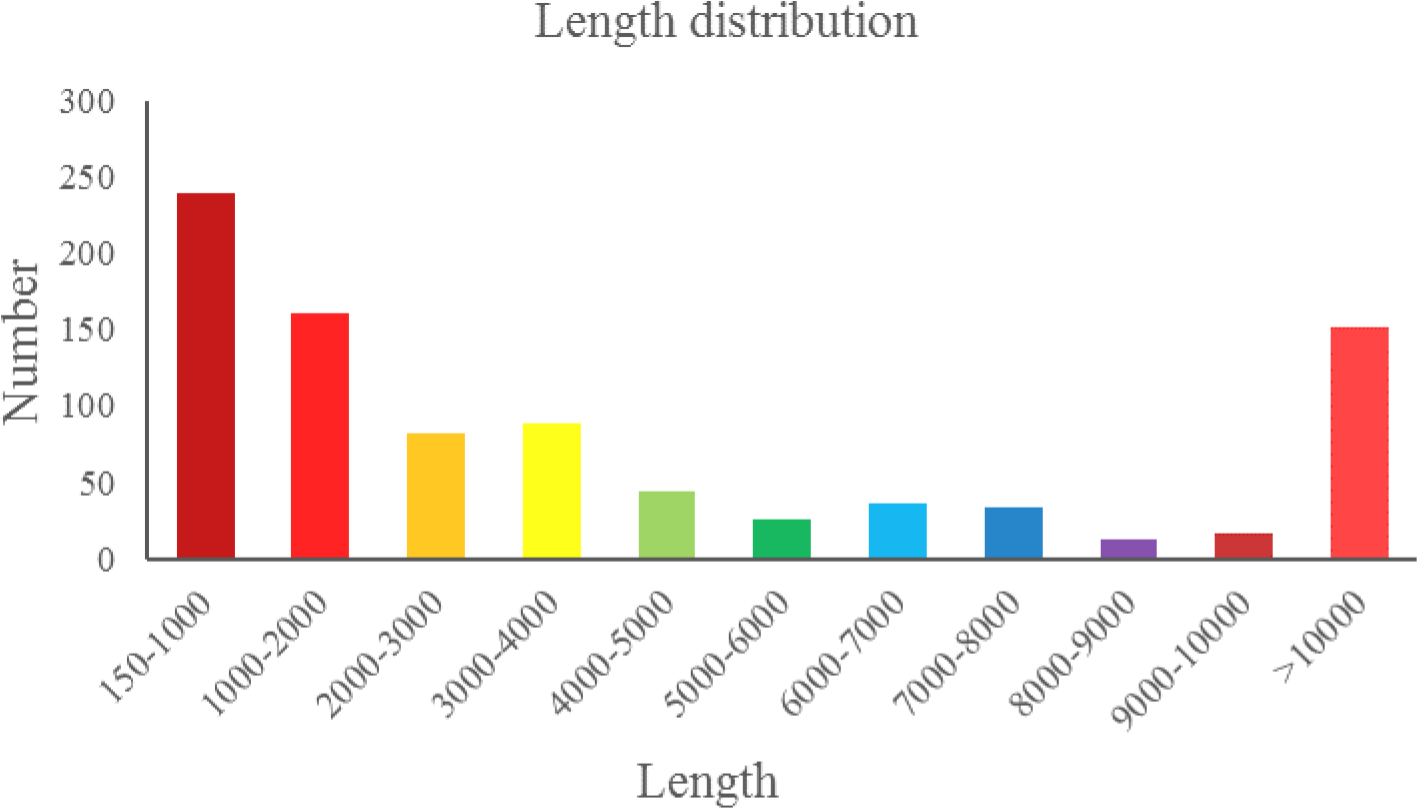
Length distribution of pear circRNAs.

To investigate the circRNAs involved in drought stress, we conducted an RNA-seq experiment. Six cDNA libraries were constructed from leaves of birch-leaf pear plants exposed to drought stress and from control plants. After removing adaptors and primer sequences, as well as short low-quality sequences, we obtained 854,138,996 clean reads in total. The Q20 and Q30 scores were all greater than 90% (Table 1), indicating that the quality of sequencing was high. After analysis of the sequences, 889 non-redundant circRNAs with an average length of 5,375 bp were obtained (S2 File), of which 614 (69.07%) ranged in length from 150 to 5,000 bp, 124 (13.95%) ranged in length from 5,000 to 10,000 bp, and all others were greater than 10,000 bp in length (Fig 1). Clean read have been deposited in the National Center for Biotechnology Information (NCBI) database and are accessible through the accession number, SRP 150567.

**Table 1:** Sequencing data for six libraries obtained by RNA-seq.

Sample	Clean reads	Clean Bases	Q20	Q30	GC content
48h-1	99947264	14.99G	97.57	93.84	43.02
48h-2	95718804	14.36G	97.56	93.45	43.60
48h-3	92290956	13.84G	97.47	93.24	43.35
0h-1	101335652	15.2G	97.61	93.56	43.19
0h-2	90096636	13.51G	95.63	93.04	44.27
0h-3	87193746	13.08G	95.00	93.32	43.49

**Fig 2:**
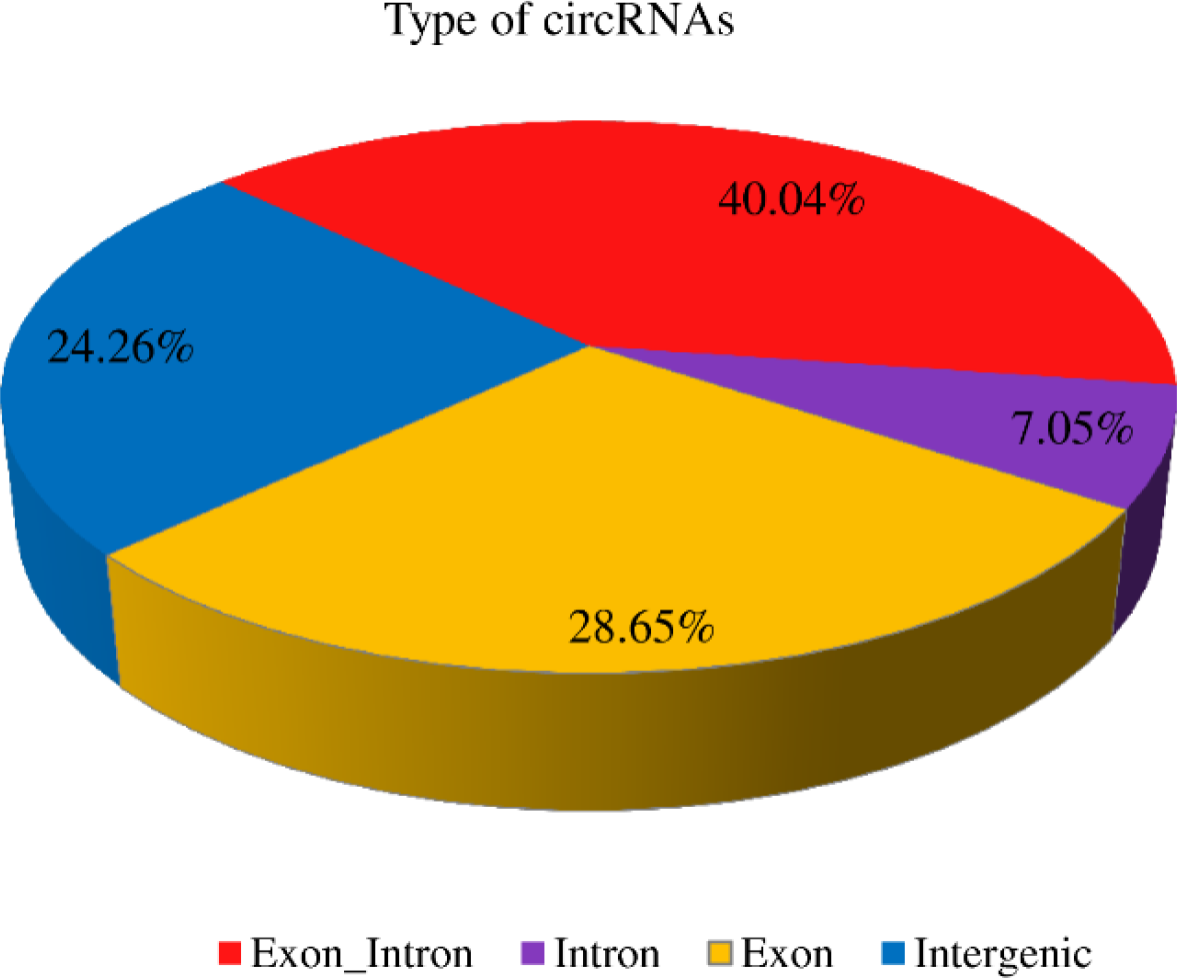
Percentages of the four types circRNAS.

Among the identified circRNAs, 186 (28.65%)were generated from exons (exonic circRNAs) (Fig 2), 173 (24.26%) were intergenic, 70 (7.05%) were generated from introns, and the remainder were exon_intron.

### CircRNAs differentially expressed in response to drought stress treatment

**Fig 3:**
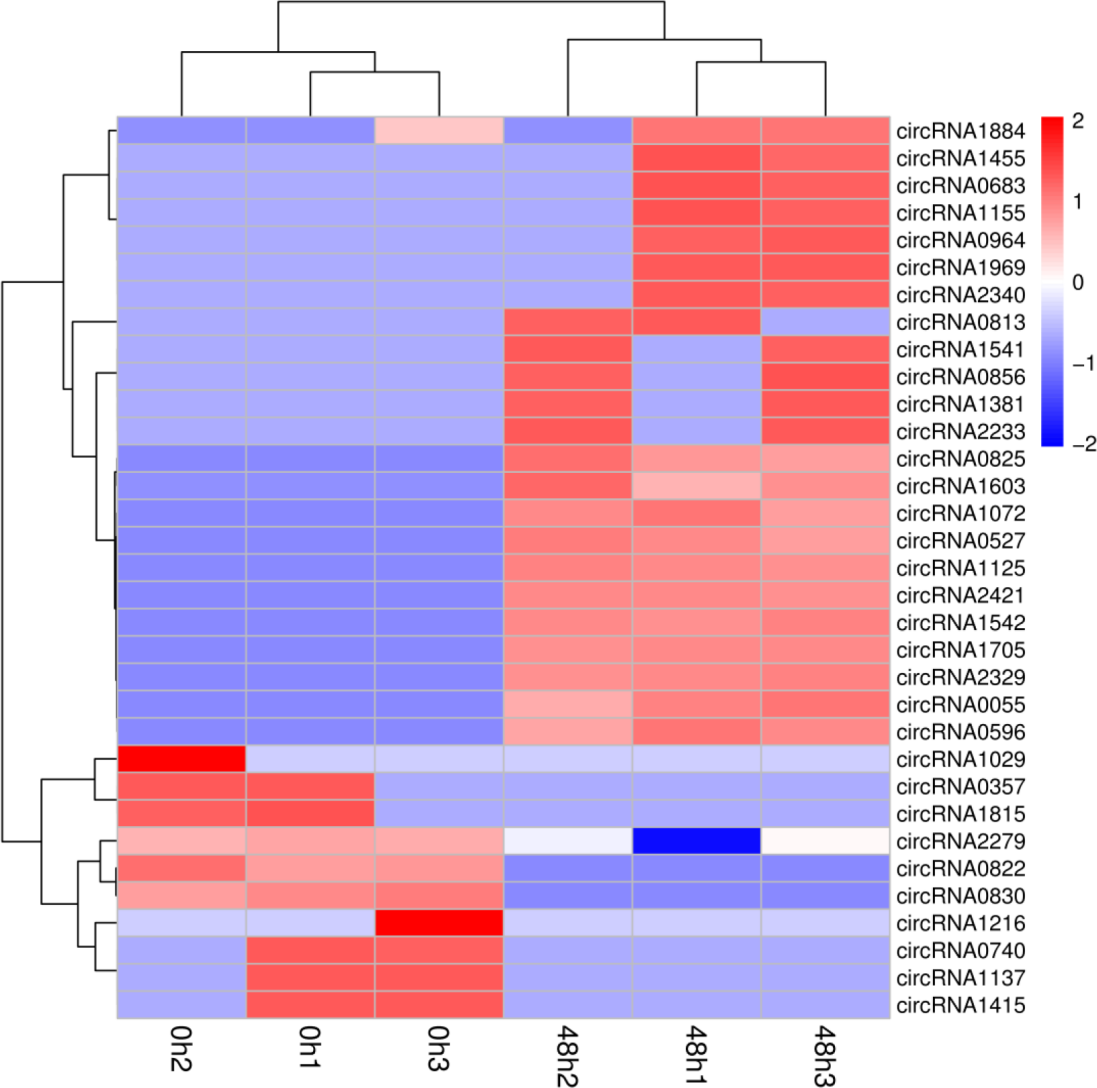
Heat map of differentially expressed circRNAs under drought stress compared with control.

The expression pattern of a gene can be used as an indicator of its putative biological function. To determine which of the circRNAs were differentially expressed between the control and treatment groups (three biological replicates per condition), circRNAs were filtered based on statistical thresholds (FDR ≤ 0.05 and 〡log2 (ratio)〡≥ 1). 33 circRNAs was found to be expressed at significantly different levels in the treatment group compared to the control group. 10 genes were downregulated under drought stress, while 23 were upregulated (S3 File). The differentially expressed genes were visualized using a heatmap (Fig 3).

### Functional analysis of circRNAs involved in drought stress

**Fig 4:**
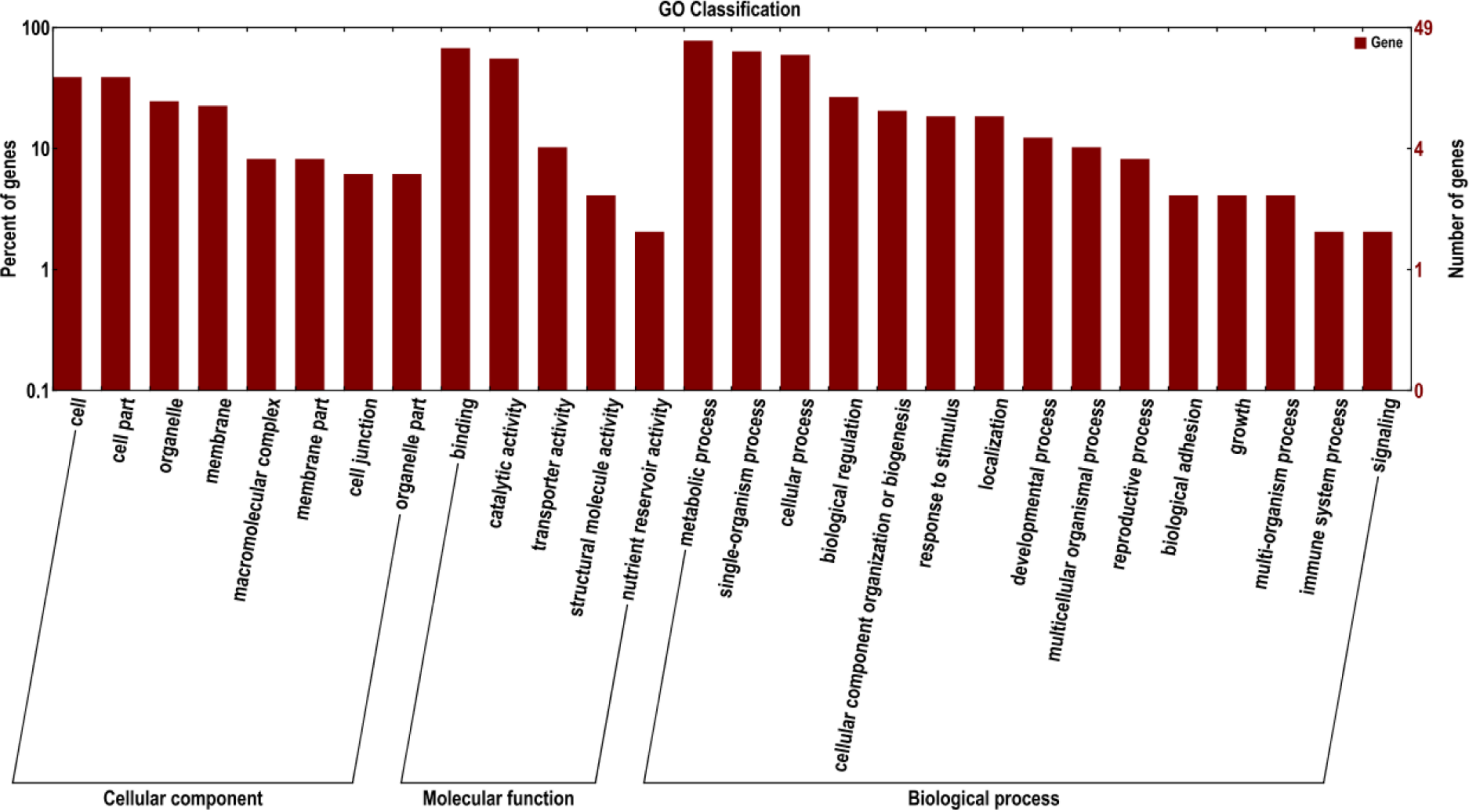
GO functional analysis of the differentially expressed circRNA’s host genes.

To further understand the potential function of circRNAs, we predicted and analyzed their host genes. The host genes of 33 differentially expressed circRNAs were categorized into 171 functional groups (S4 File), clustered into three main GO classification categories (“biological process”, “cellular component”, and “molecular function”), which contained 100, 18, and 53 functional groups, respectively. Oxidation reduction process (GO: 0055114) consisted of 11 genes was dominant in the Biological process category. Plasma membrane (GO: 0005886) with 6 genes was dominant in the Cellular component. The top GO enrichment analysis result showed in Fig 4.

**Fig 5:**
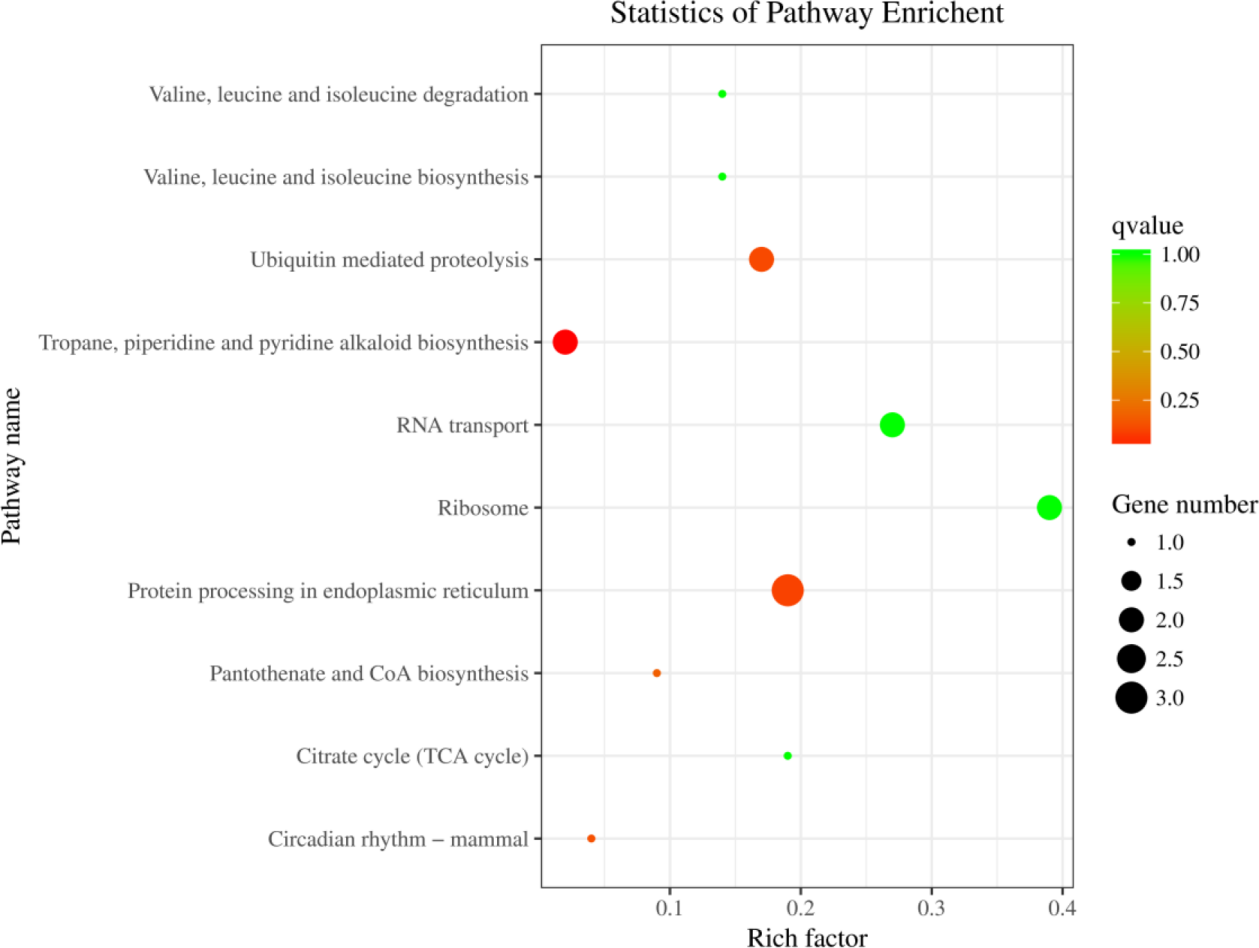
KEGG analysis of differentially expressed circRNA’s host genes.

We further analyzed the host genes of differentially expressed circRNAs for KEGG pathway enrichment and annotated them based on their involvement in 10 pathways (S5 File). Among these pathways, three host genes were assigned to “metabolic process”, while other pathways were associated with one or two genes (Fig 5). The most common pathways included “biosynthesis of secondary metabolites”, “ribosome”, “protein processing in endoplasmic reticulum”, “ubiquitin mediated proteolysis”, “RNA transport”.

### The target miRNAs of circRNAs

**Fig 6:**
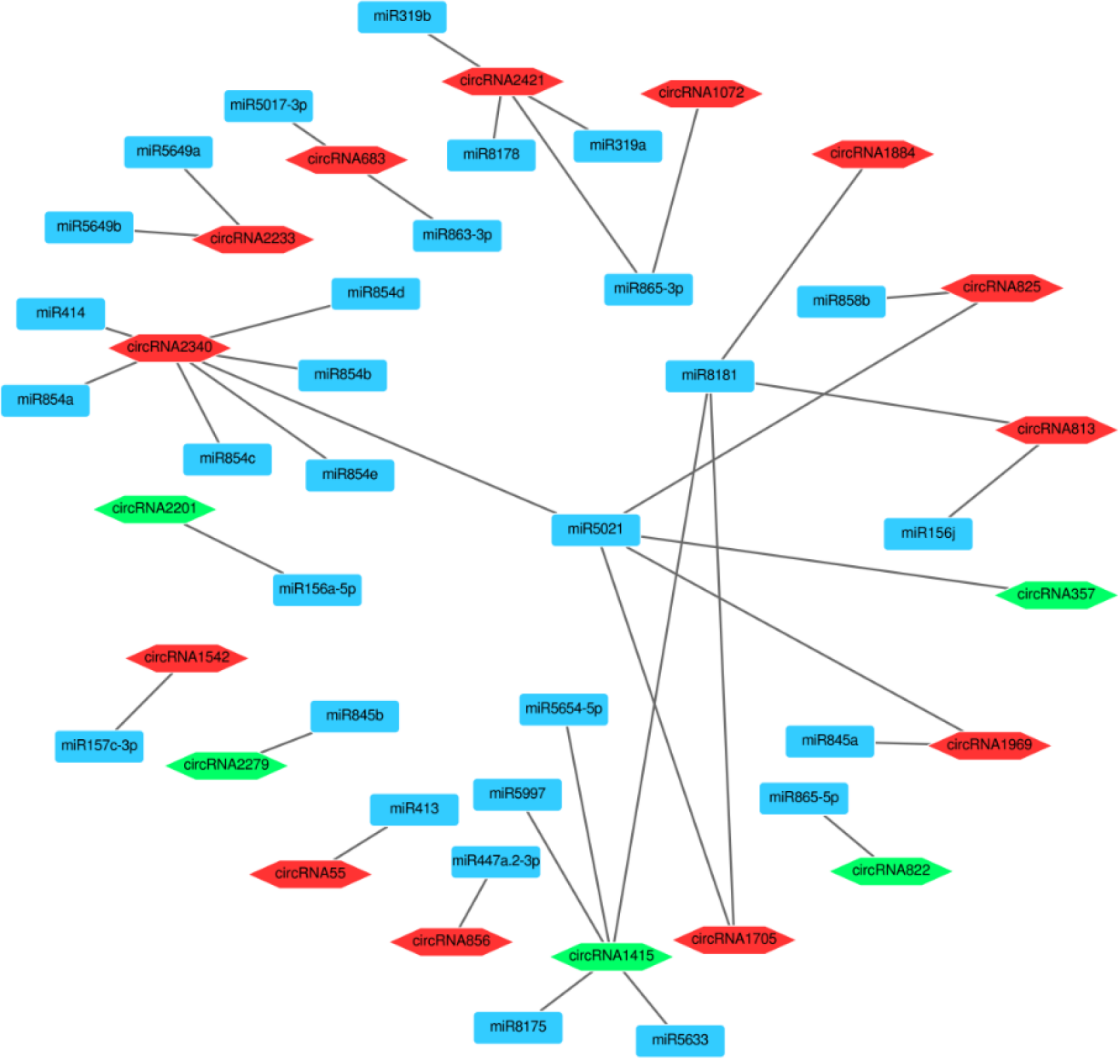
The network of differentially expressed circRNAs and their target miRNA.

Recent studies have demonstrated that circRNAs can bind miRNAs to prevent them from targeting mRNAs, thereby regulating gene expression [13]. To identify the pear circRNAs that target miRNAs, we used psRobot software to predict potential miRNA binding sites. In total, 309 circRNAs were predicted to act as sponges to a corresponding 180 miRNAs (S6 File). Among these 309 circRNAs, 146 had more than two miRNA binding sites. This implied that these circRNAs could serve as miRNA sponges. circRNA322 had 15 miRNA binding sites: miR5658, miR169a-5p, miRNA169b-5p, miR169c, miR169d, miR169e, miR169f-5p, miR169g-5p, miR169h, miR169i, miR169j, miR169k, miR169l, miR169m, and miR169n. This demonstrated that a single circRNA can target various miRNAs, and that a single miRNA can be targeted by different circRNAs. For example, miR414 could be targeted by 20 circRNAs, and miR-156 could be targeted by 52 circRNAs. The potential target miRNAs of 33 differentially expressed circRNAs are partially shown in Fig 6.

### RT-qPCR validation

**Fig 7:**
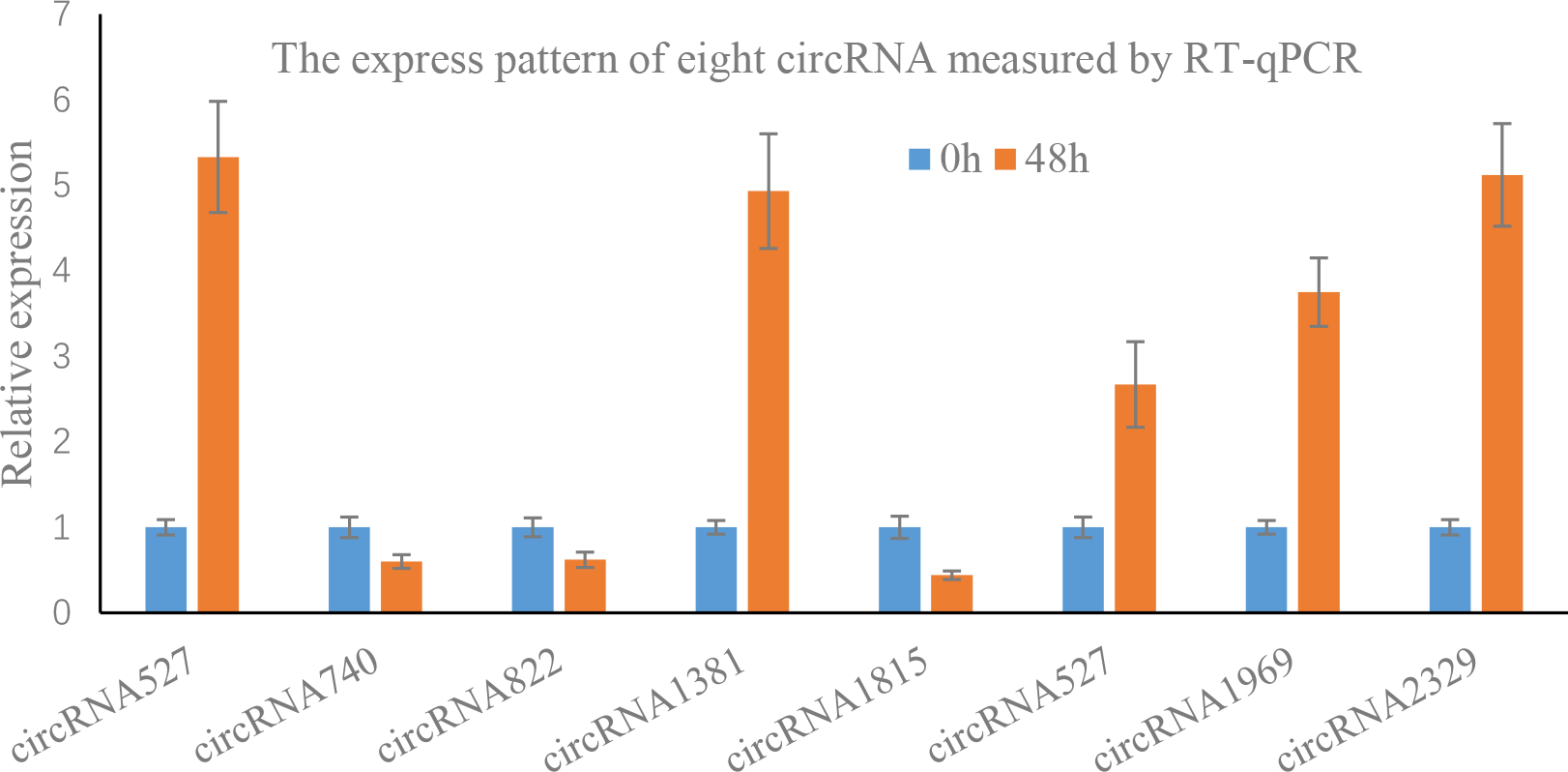
Relative expression of eight selected circRNAs measured by RT-qPCR.

To validate the reliability of the transcriptome gene expression profiles, 8 differentially expressed circRNAs were randomly selected for expression analysis through RT-qPCR (Fig 7). The expression patterns shown in the RT-qPCR results were consistent with the RNA-seq results. For example, the relative expression of circRNA527 was increase after drought stress, but the expression of circRNA822 was decreased, this was consistent with the RNA-seq result. Suggesting that the results of this experiment data analysis were reliable.

## Discussion

In the past year, circRNAs were considered to be RNA splicing errors [31]. Recent studies had identified a large number of circRNAs in mammals, and demonstrated that natural circRNAs play an important role in biological and developmental processes in animals [10, 32]. However compared with animal circRNAs, little attention has been given to plant circRNAs [17, 33]. Here, we reported the first identification and characterization of circRNAs in *pyrus betulifolia*, we obtained 889 circRNAs with an average length of 5,375 bp in total. Among other plants, 6,012, 12,037, 854, 113, and 496 circRNAs were previously identified in *Arabidopsis* [18], *Oryza sativa* [17], tomato [14], *Setaria italic*, and *Zea mays* [34], respectively. The number of circRNAs we identified in pear was greater than have been identified in tomato, *S*. *italic*, and *Z*. *mays*, but fewer than in *Arabidopsis* and *O*. *sativa*. In *Arabidopsis* and *O*. *sativa*, the cDNA libraries were generated from multiple tissue types; conversely, the cDNA libraries in the present study were generated only from leaves. Therefore, we likely identified only a subset of the total circRNAs in birch-leaf pear.

As circRNAs do not have 5ʼ and 3ʼ ends, the associated inherent stability makes them strong candidates for maintaining homeostasis in the face of environmental challenges [35]. circRNAs also often follow tissue- and stage-specific expression patterns [11,36]. In humans, all the circular transcripts to data are expressing at low levels compared to the dominant canonical linear isoform [37]. The members of a subset of circRNAs were significantly upregulated in the brain tissue of old versus young mice, whereas some were downregulated [38]. circRNAs have also been shown to be highly abundant and dynamically expressed in animal brains [39], nonalcoholic steatohepatitis samples [40], vascular cells [41], *Arabidopsis* under various stresses [42], and in various rice tissues [12]. In this study, we showed that 33 circRNAs were differentially expressed under dehydration stress and may therefore play important roles in drought-stress tolerance in pear. The apparent regulation of circRNAs appears to be a general phenomenon.

The response to drought stress in plants is a complicated process, involving several genes and metabolic network, hormones synthesis is one of important factor [34]. 2 host genes were associated with response to stress. Oxidation-reduction processed pathway is another important pathway on drought stress, in this study, a total of 11 host genes associated with this pathway. we also identified 1 host genes linked with ubiquitin, which may take part in signal transduction and the degradation of protein in response to stress [43]. “metabolic process” was the dominant subcategory in the 100 subgroups related to “biological process”. This results consistent with those of previous research [44, 45]. This provided further insight into the role of circRNAs in the drought response.

CircRNA can serve as competing endogenous RNA to bind miRNAs. In mammals, ciRS-7 has over 70 potential miRNA binding sites for miR-7. In tomato, 61 circRNAs functioned as sponges for 47 miRNAs [46]. In our study, 309 circRNAs were predicted to act as sponges for 180 miRNAs. We can therefore infer that various circRNAs containing common miRNA binding sites might act as miRNA sponges to regulate the response to drought stress in pear.

In summary, we identified 889 circRNAs in birch-leaf pear, among which 33 circRNAs were shown to be dehydration-responsive. Functional analysis showed that differentially expressed circRNAs were involved many dehydration-responsive processes, such as metabolic pathways, protein processing in the endoplasmic reticulum, and biosynthesis of amino acids. A circRNA-miRNA co-expression network indicated that the circRNAs were involved in drought-responsive processes. Our results provide a rich genetic resource for the discovery of genes related to drought stress, and can readily be applied to other fruit tree species.

## Supporting information

S1 File The primers sequences used in RT-qPCR analysis. (DOCX)

S2 File The sequences of the 889 non-redundant circRNAs of *Pyrus betulifolia* Bunge under drought stress. (FA)

S3 File The data of different expressed circRNAs of *Pyrus betulifolia* Bunge under drought stress. (XLS)

S4 File GO annotation of the host genes of different expressed circRNAs of *Pyrus betulifolia* Bunge under drought stress. (XLSX)

S5 File KEGG annotation of the host genes of different expressed circRNAs of *Pyrus betulifolia* Bunge under drought stress. (XLS)

S6 File The list of circRNAs and their target miRNA of *Pyrus betulifolia* Bunge under drought stress. (TXT)
