# Supplementary material for: Identification and characterization of circRNAs in Pyrus betulifolia Bunge under drought stress
Source: PLoS One. 2018 Jul 17;13(7):e0200692. doi: 10.1371/journal.pone.0200692 (PMC6049930; doi:10.1371/journal.pone.0200692)
Supplement: S1 File — (DOCX) [file pone.0200692.s001.docx]

Additional file1. The primers sequences used in RT-qPCR analysis

| Gene | Forward Primer | Reverse Primer | Amplicon Length(bp) |
| --- | --- | --- | --- |
| CircRNA527 | CGTCAGAAGCTAGTGCT | AATGTTAGATTGGTAAC | 117 |
| CircRNA740 | CGGTTCCATGCATTGCAC | ATCCCATGGATCCATGTT | 122 |
| CircRNA822 | CATCCATTGACAACATT | TGTGGCATCTTATGACAC | 113 |
| CircRNA1381 | GTTGTGTACGTGAGGCC | ATCTTCGTTCGGTGCCTC | 119 |
| CircRNA1815 | CCTGGAGTTCCAGACAT | ACAATTAGCATCAACCG | 119 |
| CircRNA527 | GATCGGGACCCTCATGTA | GGATTGCT GATACTGTTG | 97 |
| CircRNA1969 | CGGTGGACTCGAAGGCG | AATCGTCTCCTCCTGGTA | 109 |
| CircRNA2329 | ATGTGCACCGGTGGTCG | TGCTGCATAGGTGGTCC | 131 |
| GAPDH | TGACGTGTACGAGCTAG | GCATCACAGGATCTCAAC | 105 |
